# Is *Stevia rebaudiana* Bertoni a Non Cariogenic Sweetener? A Review

**DOI:** 10.3390/molecules21010038

**Published:** 2015-12-26

**Authors:** Gianmaria Fabrizio Ferrazzano, Tiziana Cantile, Brunella Alcidi, Marco Coda, Aniello Ingenito, Armando Zarrelli, Giovanni Di Fabio, Antonino Pollio

**Affiliations:** 1 Department of Neuroscience, Reproductive and Oral Sciences, Section of Paediatric Dentistry, University of Naples, Federico II, Naples 80131, Italy; tizianacantile@yahoo.it (T.C.); brunella.alcidi@gmail.com (B.A.); marcocoda@fastwebnet.it (M.C.); ingenito@unina.it (A.I.); 2 Bambino Gesù Hospital, Division of Dentistry and Orthodontics, Rome 00165, Italy; 3 Department of Chemical Sciences, Complesso Universitario di Monte Sant’Angelo, Cupa Nuova Cintia, 21-80126-Napoli, University of Naples, Federico II, Naples 80126, Italy; armando.zarrelli@unina.it (A.Z.); giovanni.difabio@unina.it (G.D.F.); 4 Inter-University Consortium “SannioTech”, Apollosa (BN) 82030, Italy; 5 Department of Biology, Complesso Universitario di Monte Sant’Angelo, Cupa Nuova Cintia, 21-80126-Napoli, University of Naples, Federico II, Naples 80126, Italy; antonino.pollio@unina.it

**Keywords:** *Stevia rebaudiana* Bertoni, dental caries, sweetener

## Abstract

*Stevia rebaudiana* Bertoni is a small perennial shrub of the Asteraceae (Compositae) family that is native to South America, particularly Brazil and Paraguay, where it is known as “stevia” or “honey leaf” for its powerful sweetness. Several studies have suggested that in addition to their sweetness, steviosides and their related compounds, including rebaudioside A and isosteviol, may offer additional therapeutic benefits. These benefits include anti-hyperglycaemic, anti-hypertensive, anti-inflammatory, anti-tumor, anti-diarrheal, diuretic, and immunomodulatory actions. Additionally, critical analysis of the literature supports the anti-bacterial role of steviosides on oral bacteria flora. The aim of this review is to show the emerging results regarding the anti-cariogenic properties of *S. rebaudiana* Bertoni. Data shown in the present paper provide evidence that stevioside extracts from *S. rebaudiana* are not cariogenic. Future research should be focused on *in vivo* studies to evaluate the effects on dental caries of regular consumption of *S. rebaudiana* extract-based products.

## 1. Introduction

In the United States, dental caries is the most common chronic disease in children, and it is increasing in prevalence among two- to five-year-olds [1,2]. Nevertheless, caries prevalence varies from population to population. In developing countries, such as the Philippines, an early childhood caries prevalence of up to 85% has been reported for disadvantaged groups [3]. In the Western world, studies have reported that the prevalence of dental caries at three years of age was 15.4%, but strong associations were found with socioeconomic status and ethnicity [4,5].

A significant improvement in dental caries levels worldwide may be attained by the implementation of caries preventive strategies, paying special attention to high-risk groups that are represented by people with low economic-social status in developed countries and people who lives in less developed countries [6,7].

Therefore, current research is focused on the elaboration of a new methodology against dental caries that is based on the identification and the characterization of natural active compounds that have anti-caries activity, reduce cariogenic microflora pathogenicity and/or remineralize dental hard tissues [8,9,10,11,12].

## 2. Dental Caries Pathogenesis and Its Relation with Alimentary Factors

Sugars are recognized as the most important dietary factor in the development of dental caries. Furthermore, the role of acid fermented products of sugars in enamel dissolution by the action of bacteria, such as *Streptococcus mutans* and *Lactobacillus casei* [13], is clear. Upon fermentation by oral bacteria, sucrose molecules are transformed into energy and high quantities of acidic substances that increase the concentration of hydrogen ions, by lowering the pH, and dissolve enamel, the cementum, and dentin [14]. Thus, frequent exposure to this carbohydrates creates conditions for caries onset by promoting demineralization.

As an additional virulence mechanism, cariogenic bacteria populating the dental biofilm produce exopolysaccharides that are able to create a protective environment against the physiological antibacterial mechanisms of the mouth [15].

Since these microorganisms are the most prominent factors in the dental caries process, modifying their metabolism by reducing the production of lactic acid in the oral cavity will provide additional justification for the prevention of dental caries [16]. Numerous studies show that the form in which sugars are ingested and the frequency of their consumption are directly related to the prevalence of caries [17]. Sugars also contribute to the increase in an elevated number of health problems such as obesity and dysmetabolic diseases [18,19].

### 2.1. Development of Natural Sweeteners in Food Factory Research

As mentioned previously, there is a definite relationship between the dietary consumption of sucrose and the incidence of dental caries. Therefore, intensive research for a low calorie, non-cariogenic sweetener has been performed to provide an alternative compound to sugar for use in food and drugs, and several artificial sweeteners have been introduced by the food industry to sweeten food and beverages. Research for alternatives to sucrose have resulted in the development of synthetic sweeteners, many of which are considered safe for teeth, such as aspartame, saccharin, cyclamate, xylitol, and mannitol [17]. These sweeteners have also been used as sugar substitutes for caries-active patients.

However, in addition to their benefits, animal studies have proven that artificial sweeteners cause weight gain, brain tumours, bladder cancer, and many other health hazards [17]. Thus, research must continue to identify natural foods and components that protect against dental caries, particularly those that have practical dietary application and can help make dietary advice effective.

The ideal product would be calorie-free, non-carcinogenic, non-mutagenic, not heat degradable, economical to produce, and it would provide sweetness with no unpleasant aftertaste. However, obtaining these properties in a single product has been extremely challenging.

Since ancient times, active compounds from plant origins have been used as medicines for various diseases and microbial infections.

Recent research has also been oriented towards the discovery and evaluation of novel, potentially non-cariogenic, sweeteners from nature. Several highly sweet plant constituents are commercially utilized in foods and beverages as non-cariogenic sucrose substitutes in Japan and in some other Asian, European, and American countries, including the diterpene glycoside, stevioside, a steviol glycoside extracted from *Stevia rebaudiana* (Bertoni).

### 2.2. The Genus Stevia: Botanical and Ethnobotanical Remarks, with an Emphasis on S. rebaudiana Bertoni

*Stevia* Cav. is a genus of herbaceous and shrubby plants distributed exclusively in the American Continent, from the Southern United States to Central and South America. More than 200 species belong to this genus, which is taxonomically placed in the Tribe Eupatoriae of the family Asteraceae [20]; most species are distributed between Mexico (approximately 100) and Brazil (approximately 40). The members of *Stevia* occupy a range of habitats, from mountain forests (above 1000 m) to the borders of rivers and dry valleys [21], preferring semi-humid and cold climates [22]. *Stevia* species present a very similar morphology of inflorescence, which is composed of five small tubular flowers with numerous hairs on the inner surface of the white corollas.

**Figure 1:**
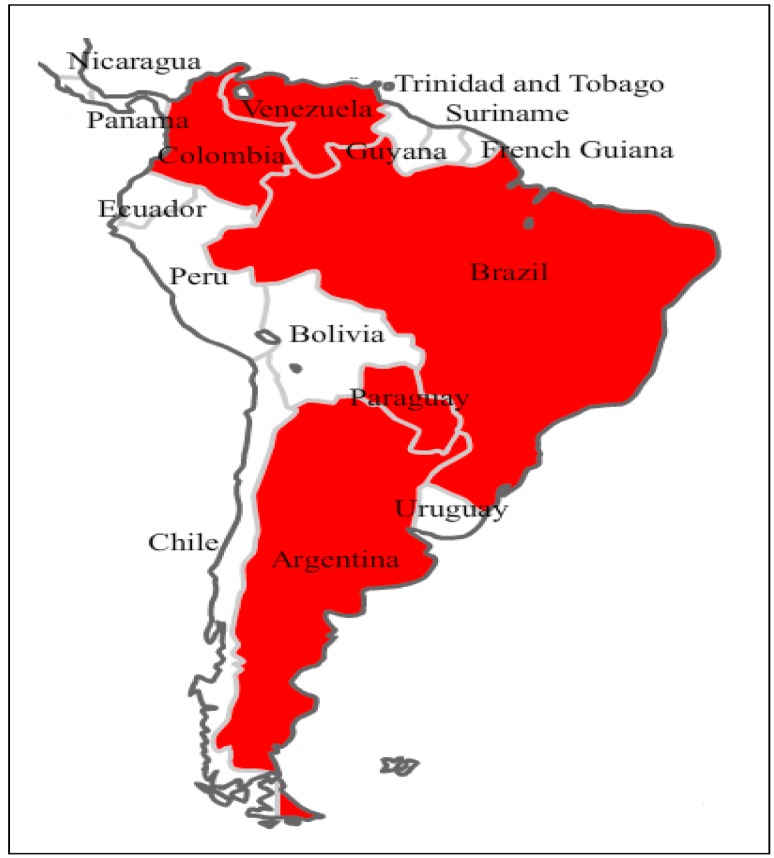
Countries of South America where *S. rebaudiana* grows spontaneously.

**Figure 2:**
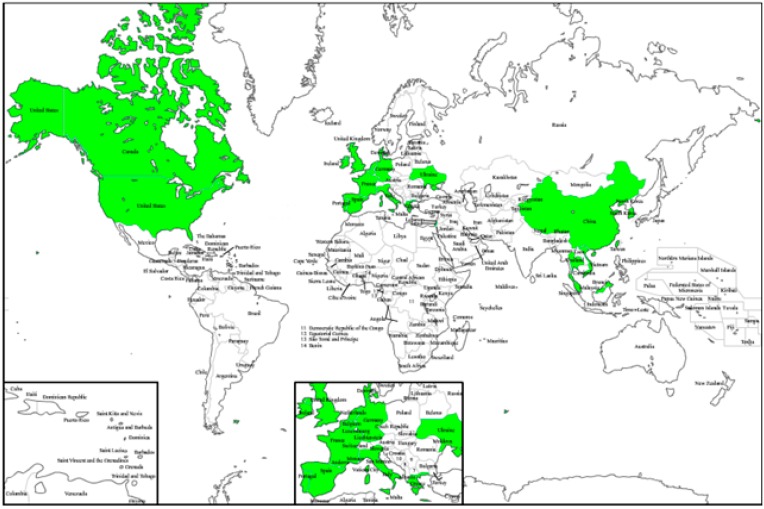
Regions of the world where it is possible to cultivate *S*. *rebaudiana*.

In Central and South America, numerous *Stevia* species, such as *S. salicifolia* Cav. and *S. lucida* Lag., have long been known for their ethnopharmacological uses, ranging from anti-helminthic to anti-rheumatic and anti-inflammatory applications. Certain species are also used as an emetic (*S*. *rhombifolia* HBK), for the treatment of cardiac conditions (*S. cardiatica* Perkins) or as anti-diarrheal (*S. balansae* Hieron, *S. trifida*), whereas diuretic properties have been attributed to *S. eupatoria* (Spreng.) Willd. and *S. pilosa* Lag. [23]. Apparently, *S. rebaudiana* (Bertoni) Bertoni, which originated from Northeastern Paraguay, is a unique species containing the glycosides stevioside and rebaudioside A, responsible for the sweet taste of the leaves [24]. It is a perennial shrub, spontaneously growing in the subtropical, mesothermal and humid habitats of South America (Figure 1) [25]. The plant is rhizomatous, with a well-developed root system; the stem is erect and woody, with tiny hairs in the lower part; and the slightly pubescent leaves are sessile, with a dentate margin in the upper part of the plant and entirely at the bottom. *S. rebaudiana* is not common in its type locality, but it is cultivated at least in three continents (Figure 2), particularly in Asia, from China to Thailand [26].

In Paraguay, *S. rebaudiana* flowers from January to March, whereas in the Northern Hemisphere, the flowering time is between September and December. In its native habitat, the plant occurs mainly in sandy soils; under cultivation, however, the growth and flowering of *S. rebaudiana* depend on solar radiation, photoperiod, temperature and water availability of the soil [27]. When cultivated under long-day conditions, *S*. *rebaudiana* increases the foliar area and the concentration of sweetening glycosides in the leaves [28]. *S. rebaudiana*, often referred to as *the sweet herb of Paraguay*, has been widely used in many countries, including China, Japan, Korea, Brazil, and Paraguay, either as a substitute for sucrose in foods and beverages or as a household sweetening agent [29]. The plant is rich in carbohydrates (62% dry weight, dw), protein (11% dw), crude fibre (16% dw), minerals (K, Ca, Na, Mg, Cu, Mn, Fe, Zn), and essential amino acids [30].

### 2.3. S. rebaudiana Chemical Constituents and Extraction Procedures

The extracted active ingredient of *S. rebaudiana* is a white crystalline substance, and it has been used for centuries to sweeten food and beverages by the indigenous people of South America.

The compounds responsible for the natural sweetness of *S. rebaudiana* leaves include diverse diterpenoid glycosides derived from a steviol skeleton. These steviol glycosides also exhibit low calorific value, which is interesting for promising therapeutic applications, particularly for the treatment of disturbances in sugar metabolism.

Recently, several modern techniques have been introduced for the extraction of commercially-important stevioside metabolites from the plant. These methods were optimized in terms of temperature, duration of the process, stability and quantity of molecules extracted, whereas the conventional methods of extraction (Soxhlet extraction, hot or cold maceration, and hydro distillation) were less desirable from both an economic and an extractive standpoint (Table 1) [31,32,33].

**Table 1:** Different extraction methods of steviosides from air dried crushed leaves of *S*. *rebaudiana*.

Extraction Method	Problems/Advantages/Disadvantages	Ref.
Water extraction process	Optimization in terms of pH, temperature, pressure, duration.	[31]
Water leaching	Time and labor consumption.	[32]
Ethanol leaching	More rapid than water leaching, keeping the same extraction recovery.	[32]
Supercritical fluid extraction (SFE)	Low solvation power for polar stevioside, need to use a co-solvent. Optimization in terms of pressure and temperature.	[32]
Pressurized fluid extraction (PFE)	Multiple rate, lower solvent consumption, enhanced mass transfer and increased solvation power. Limited to thermally stable analytes.	[32]
Pressurized hot water extraction (PHWE)/subcritical water extraction	Extraction of nonpolar compounds with water, environmentally-friendly and economically-beneficial alternative to harmful organic solvents.	[32]
Microwave assisted extraction	Optimization in terms of solvent and solvent volume. Extraction of compounds of commercial importance in less time, with lesser solvent and causing comparatively lesser harm to the environment.	[33]
Soxhlet extraction	Optimization in terms of time, solvent, and solvent volume.	[33]
Cold maceration	Optimization in terms of time, solvent, and solvent volume.	[33]

Microwave-assisted extraction uses pressurized and/or supercritical fluids or microwaves to reduce extraction time. Moreover, in microwave assisted extraction, the typical solvent volume used ranges from 10 to 30 mL per gram of plant sample, much less than that required in conventional extraction methods (Table 1) [33].

Steviosides are soluble in water, the optimal solvent, and water is the most efficient polar solvent in regard to its dielectric constant to absorb microwave energy, dissipate it in the external environment and finally promote the exudation of steviosides from the cells of leaves. Methanol and water can be used for extraction under pressure as well. Methanol is the best solvent in the temperature range of 110 to 160 °C [32]. Another important aspect is the time required for the extraction, which can vary from 16 to 24 h for Soxhlet extraction to just minutes, possibly longer, with a microwave assisted extraction.

The leaves have a stevioside contents between 4% and 20% of their dry weight, in according on the cultivar and growing conditions. The maximum amount was observed in microwave assisted extraction of *S. rebaudiana* leaves, while the minimum was obtained by cold maceration of the leaf powder, using water as the solvent for both methods [33].

The three major constituents of the leaf extract of *S. rebaudiana* were stevioside, rebaudioside A, and rebaudioside C (from 3% to 17%, by weight) [34,35]. Other compounds present at lower concentration are: steviolbioside, rebaudiosides B, D, E, F, and steviolmonoside [36,37].

**Figure 3:**
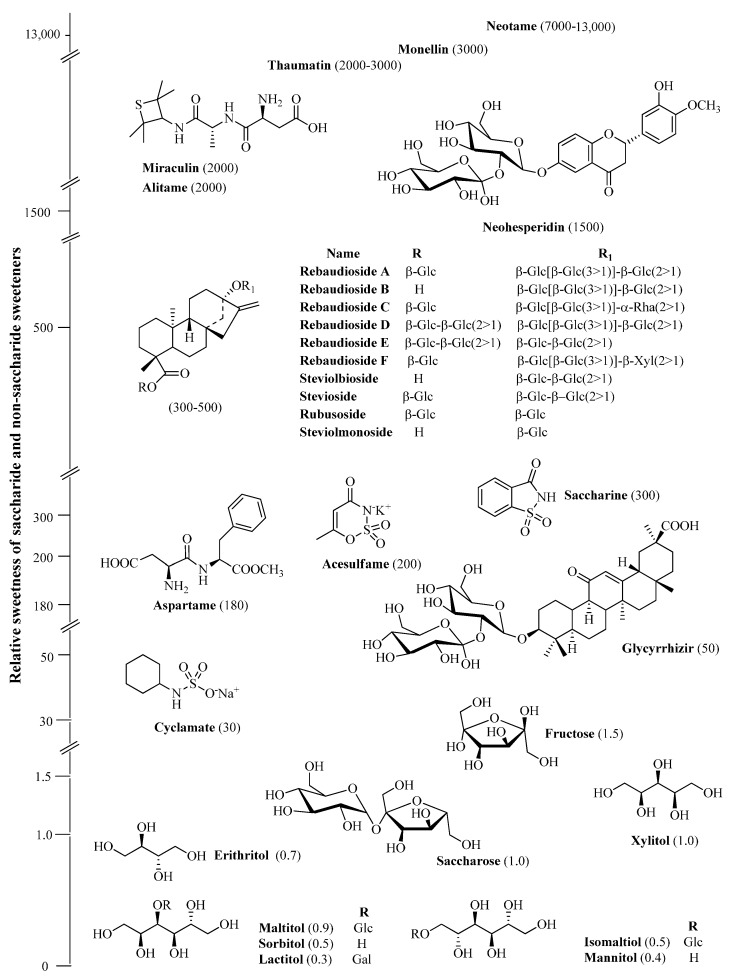
Sweetness of the most common artificial and natural sweeteners.

Stevioside, the main sweet component in the leaves of *S. rebaudiana* (Bertoni) Bertoni tastes approximately 300 times sweeter than sucrose. The structures of the sweet components of *S. rebaudiana*, which occur primarily in the leaves, are provided in Figure 3.

Isolated steviosides can be purified using various methods including column chromatography, TLC and HPLC methods. Finally the isolated compounds were analyzed and characterized using analytical methods such as UV, FTIR, MS, and NMR analyses.

### 2.4. Medicinal and Alimentary Uses of S. rebaudiana Glycosides

There are three types of *S. rebaudiana*-based products: the regular products, which consist mainly of a stevioside; the Reva A products, which consist mainly of rebaudioside A; and the sugar metastasis product. In the regular products, the content ratio of stevioside to rebaudioside ranges from 7:3 to 8:2, while in Reva A, this ratio is approximately 1:3. Since rebaudioside has a very sweet taste, the quality of sweetness for Reva A products is higher than regular ones [38]. Steviosides offer several advantages over other non-caloric sucrose substitutes: they are heat-stable, resistant to acid hydrolysis and non-fermentable [39].

Further studies have suggested that in addition to sweetness, steviosides and their related compounds, including rebaudioside A and isosteviol (a metabolic component of stevioside), may also offer therapeutic benefits. These benefits include: anti-hyperglycaemic, anti-hypertensive, anti-oxidant [40], anti-tumor [41,42], anti-diarrheal, diuretic, gastro- [43] and renal-protective [44], anti-viral [45], and immunomodulatory [46,47] actions. (Table 2 and Table 3).

**Table 2:** Medicinal uses of *Stevia* species [48,49].

Species	Medicinal Uses
*S. balansae* Hieron.	Intestinal affections
*S. cardiatica* Perkins	Cardiac affections
*S. connata* Lag.	Gastric affections
*S. elatior* H. B. K	Vulnerary, Dermatologic affections
*S. eupatoria* (Spreng.) Willd.	Renal affections
*S. glandulosa* Hook. & Arn.	Antipyretic, Cold
*S. lucida* Lag.	Anti-inflammatory, Anti-dolorific, Flue, Vulnerary
*S. macbridei* B. L. Rob.	Gynecologic affections
*S. nepetifolia* H. B. K.	Gynecologic affections
*S. pilosa* Lag.	Antipyretic, Intestinal affections, Renal affections
*S. plummense* A. Gray	Vulnerary
*S. puberula* Hook.	Gastric affections
*S. rebaudiana* (Bert.) Bertoni	Gynecologic affections, Anti-diabetic
*S. rhombifolia* Kunth	Gastric affections
*S. salicifolia* Cav.	Anti-rheumatic, Intestinal affections, Cold
*S. serrata* Cav.	Vulnerary, Anti-cough
*S. subpubescens* Lag.	Anti-dolorific, Gynecologic affections, Gastric affections
*S. trifida* Lag.	Gastrointestinal affections

**Table 3:** Medicinal uses of *Stevia* species.

Medicinal Properties	Ref.
Anti-hyperglycemic/Anti-diabetic	[40]
Anti-Inflammatory	[47]
Antimicrobial	[41]
Antioxidant	[40]
Antitumor	[42]
Antiviral	[45]
Immunomodulatory	[46]
Gastroprotective activity	[43]
Renal protective	[44]

Fengyang *et al.* [50] examined the anti-inflammatory proprieties of stevioside and discovered that stevioside exerts its anti-inflammatory effect by inhibiting the activation of NF-κB and mitogen-activated protein kinase signaling and the release of pro-inflammatory cytokines (Table 2 and Table 3).

The effects of stevioside and its metabolite, steviol, on human colon carcinoma cell lines were studied from Boonkaewwan *et al.* [51] in 2008. Their results demonstrated two biological effects of steviol in the colon: the stimulation of Cl(−) secretion and the attenuation of TNF-alpha stimulated IL-8 production.

The anti-hyperglycaemic and blood pressure-reducing effects of *S. rebaudiana* were investigated in 2003 by Jeppesen *et al.* [52] in a long-term study of type 2 diabetic Goto-Kakizaki (GK) rats. According to their results, stevioside may determine an increasing of insulin secretion, inducting genes involved in glycolysis. It can also: improve the nutrient-sensing mechanisms, rise cytosolic long-chain fatty acyl-coenzyme A (CoA), and control down-regulation of phosphodiesterase 1 (PDE1). They concluded that stevioside demonstrates a dual positive effect: both antihyperglycemic and blood pressure-lowering actions.

As mentioned above, the steviol glycoside is currently used in several countries as a sweetener, and it has been extensively tested to demonstrate that its use is safe for humans. In 2002, *S. rebaudiana* ranked second in the sales of herbal supplements in the USA.

According to the Joint FAO/WHO Expert Committee on Food Additives (JECFA, 2004), the consumption of *S. rebaudiana* has been generally regarded as safe [53].

Aqueous extracts of *S. rebaudiana* leaves have been approved since 2008 by the JECFA as sugar substitutes in many foods and beverages in the Western and Far East Asian countries. However, JECFA has requested additional information to change the temporary accepted daily intake (ADI) of 0–2 mg·kg^−1^·day^−1^ for steviol glycoside. The European Union approved stevia additives in 2011 [54].

A review of several internet databases and websites was conducted to identify literature related to the antimicrobial effects of stevioside extract and its role in caries prevention and dental health promotion. The keywords that were used individually or in combination included: dental caries, sweeteners, non-caloric sweeteners, stevia, stevioside, and antimicrobial activity.

### 2.5. Caries Prevention Activity of S. rebaudiana Extracts and Steviol Glycosides

Presently, *S. rebaudiana* is the only species of the genus with recognized antibiotic properties. The antimicrobial effects of *S. rebaudiana* have been ascribed to the presence of stevioside and related compounds, but their role in caries prevention and dental health promotion is not fully understood. In 2010, Mohire and Yadav [55] conducted a four week clinical study in patients with oro-dental problems to develop a chitosan-based polyherbal toothpaste (including *S*. *rebaudiana* extract). They also evaluated its plaque-reducing ability and efficacy in the reduction of dental pathogens using chlorhexidine gluconate (0.2% *w*/*v*) mouthwash as the positive control.

The study involved 18 subjects who were divided into three groups. The groups were treated as follows: Group-I, placebo, toothpaste without chitosan and herbal ingredients; Group-II, positive control, CHX (0.2% *w*/*v*) mouthwash; and Group-III, test (Polyherbal), toothpaste with chitosan, eugenol, and *Pterocarpus marsupium* (PM), *S. rebaudiana*, and *Glycyrrhiza glabra* aqueous extracts. Authors determined the total microbial count in order to obtain the reduction, in percentage, of oral bacterial count during the treatment period.

At the end of the study, the herbal extracts were shown to possess satisfactory antimicrobial activity against most of the dental pathogens. The chitosan-containing polyherbal toothpaste significantly reduced the plaque index from 70% to 47% and the bacterial count from 85% to 29%.

The authors concluded that chitosan-based polyherbal toothpaste represented a promising novel oral hygiene product compared with the currently available oral hygiene products. Nevertheless, in this study, the role of *S. rebaudiana* in reducing antimicrobial count is not clear: this effect, in fact, could be the result of synergic action of all active principles involved in the toothpaste.

In 2013, Giacaman *et al.* [56] investigated the cariogenic and enamel demineralization potential of several sweeteners in an artificial caries model.

Bovine enamel slabs were utilized as the culture medium for *S*. *mutans* UA159 biofilm that were exposed to different sweeteners in powder or tablet form, as including *S. rebaudiana* extracts, sucralose, saccharin, aspartame, and fructose, three times a day for five minutes The caries-positive and caries-negative controls were 10% sucrose and 0.9% NaCl, respectively. After five days, the biomass, bacterial counts, and intra- and extracellular polysaccharides of the biofilm were assessed. Surface microhardness was measured before and after the experiment to evaluate enamel demineralization, which was expressed as percentage of surface hardness loss (%SHL). The results of this study suggest less cariogenic effects and enamel demineralization for all tested sweeteners except sucrose. Compared to sucrose, *S. rebaudiana* extracts, sucralose and saccharin reduced the number of viable cells (*p* < 0.05), and all sugar alternative sweeteners reduced extracellular polysaccharide formation. Nevertheless the primary limitation of this study is that the artificial substrate does not allow a biofilm formation rate comparable with a real clinical situation.

In 2012, Gamboa and Chaves [57] evaluated the antibacterial activity of *S. rebaudiana* leaf extracts against cariogenic bacteria. They prepared extracts from dried leaves in hexane, methanol, ethanol, ethyl acetate, and chloroform, and they evaluated, using well diffusion method, the antibacterial capability of the five extracts for 16 bacterial strains of the genera *Streptococcus* (*n* = 12) and *Lactobacillus* (*n* = 4).Lactobacilli were the most *sensitive*, with an inhibition zone between 12.3 and 17.33 mm. Moreover, Blauth de Slavutzky [58] conducted an *in vivo* study to evaluate the accumulation of dental plaque after rinsing with a solution of 10% sucrose four times daily for five days and compared it to rinsing with the same frequency using a 10% solution of *S. rebaudiana* extract, which was prepared with 100 g of *stevia* boiled for 2 h in 3 L of distilled water. Consequently, it was demonstrated that *S. rebaudiana,* after rinsing, reduced dental plaque between 57%–82% less than sucrose solution, when measured by Silness-Löe index and 10%–40% less when measured by O’Leary index of plaque.

In 2014, Brambilla *et al.* [16] evaluated the effect of *S. rebaudiana* extracts on *in vitro S. mutans* biofilm formation and the *in vivo* pH of plaque. Three separate 10% solutions of stevioside, rebaudioside A and sucrose were prepared. The microbological count *in vivo* was measured using a MTT assay. Twenty volunteers rinsed with each solution for one minute and then the plaque Ph was analyzed seven times after the rinses. Higher *in vitro S. mutans* biofilm formation was observed with the sucrose solution (*p* < 0.01). After 5, 10, 15, and 30 min, the *in vivo* sucrose rinse produced a statistically significantly lower pH value compared to the *S*. *rebaudiana* extracts (F = 99.45, *p* < 0.01). Therefore, *S. rebaudiana* extracts can also be considered non-acidogenic [16].

In 1992, Das *et al.* [59] tested stevioside and rebaudioside A for cariogenicity in albino Sprague-Dawley rats. The authors divided sixty rat pups colonized with *S. sobrinus* into four groups and fed them their basal diets with added stevioside, rebaudioside A or sucrose as follows: group 1, 30% sucrose; group 2, 0.5% stevioside; group 3, 0.5% rebaudioside A; and group 4, no additional chemicals. Significant differences resulted in sulcal caries scores and *S. sobrinus* counts between group 1 and the other three groups. In fact, there was no significant difference between the stevioside, rebaudioside A and no-addition groups. Thus, neither stevioside nor rebaudioside A were cariogenic under the conditions of the study, whose primary limitation is the use of a not human sample.

Zanela *et al.* [60] investigated the effect of daily mouth-rinse use on dental plaque accumulation and on salivary *S*. *mutans* in 200 children in 2002. The solutions used were: a placebo solution composed of mentholated deionized water (group I); 0.12% chlorhexidine gluconate associated to 0.05% sodium fluoride (group II); 0.2% chlorhexidine digluconate (group III); and 0.5% stevioside mixed with 0.05% sodium fluoride at pH 3.4 (group IV). To verify the accumulation of plaque, it was assessed the Löe index method at the beginning and end of the experiment. Moreover, the analysis of cariogenic streptococci was accomplished on three saliva samples collected at three different times: before the first mouth-rinse, 24 h after the first mouth-rinse and one week after the last mouth-rinse. The mouth-rinsing routine was performed daily for 4 weeks.

The solution used by group III was the least accepted by children. Furthermore, as solution II was utilized by group II, it caused mild dental pigmentation. There were no statistically significant differences in the levels of *S*. *mutans*, most likely due to the low initial levels observed in each of the four groups (Table 4).

**Table 4:** Caries prevention activity of *S. rebaudiana* extracts and steviol glycosides.

Source	Type of Study	Results	Ref.
*S. rebaudiana* aqueous extract	*In vivo*	Reduction of plaque index by 70.47%.	[56]
*S. rebaudiana* aqueous extracts	*In vitro*	Reduction of extracellular polysaccharide formation.	[57]
*S. rebaudiana* methanol and ethanol extracts	*In vitro*	Inhibition of growth of *Lactobacilli.*	[58]
*S. rebaudiana* aqueous extracts	*In vivo*	Reduction of plaque index.	[59]
*S. rebaudiana* aqueous extracts	*In vitro* and *in vivo*	*S*. *rebaudiana* extracts are non-acidogenic.	[16]
Solution containing 0.5% Stevioside and Rebaudioside A	*In vivo*	Dental plaque reduction was not evident using stevioside mouth rinses.	[61]
Stevioside extracts	*In vitro*	Stevioside and rebaudioside A are not cariogenic.	[60]

## 3. Future Research

Despite advances made in the fluoridation of water and in the dispersal of oral hygiene standards, dental caries still remains a major health problem, affecting all countries worldwide. Because the current therapeutic strategies to prevent dental diseases are not entirely void of side effects, it should be necessary to develop new alternative approaches for clinical bacterial control. Recent research has demonstrated that plant extracts may be a suitable alternative treatment for caries [61,62,63,64,65]. Several plants products have been studied for their capability to contrast dental plaque formation, but a very limited number of these natural products has found therapeutic application, such as compounds rich in polyphenols [62].

Data shown in present review evidence that stevioside extracts from *S. rebaudiana* are not cariogenic in adult population. However, future research should be focused on *in vivo* studies to evaluate the effects of regular consumption of *S. rebaudiana* extract-based products on dental caries both in adult and pediatric population.
